# Bioenergetic Profiling of the Differentiating Human MDS Myeloid Lineage with Low and High Bone Marrow Blast Counts

**DOI:** 10.3390/cancers12123520

**Published:** 2020-11-26

**Authors:** Aikaterini Poulaki, Theodora Katsila, Ioanna E. Stergiou, Stavroula Giannouli, Jose Carlos Gόmez-Tamayo, Evangelia-Theophano Piperaki, Konstantinos Kambas, Aglaia Dimitrakopoulou, George P. Patrinos, Athanasios G. Tzioufas, Michael Voulgarelis

**Affiliations:** 1 Department of Pathophysiology, School of Medicine, National and Kapodistrian University of Athens, 115 27 Athens, Greece; aikaterini.poulaki@gmail.com (A.P.); stergiouioanna@hotmail.com (I.E.S.); kkampas@hotmail.com (K.K.); agtzi@med.uoa.gr (A.G.T.); 2 Second Department of Internal Medicine, Hematology Unit, School of Medicine, National and Kapodistrian University of Athens, 115 27 Athens, Greece; 3 Institute of Chemical Biology, National Hellenic Research Foundation, 116 35 Athens, Greece; thkatsila@eie.gr; 4 Research Programme on Biomedical Informatics (GRIB), Hospital del Mar Medical Research Institute (IMIM), Department of Experimental and Health Sciences, Universitat Pompeu Fabra, 08003 Barcelona, Spain; josecarlosgt@gmail.com; 5 Department of Microbiology, School of Medicine, National and Kapodistrian University of Athens, 115 27 Athens, Greece; epiper@med.uoa.gr; 6 Laboratory of Molecular Genetics, Department of Immunology, Hellenic Pasteur Institute, 115 21 Athens, Greece; 7 Laboratory of Flow Cytometry, Immunology Department, Laiko Hospital of Athens, 115 26 Athens, Greece; liladim1@hotmail.com; 8 Department of Pharmacy, University of Patras, 265 02 Patras, Greece; gpatrinos@upatras.gr

**Keywords:** myelodysplastic syndrome, metabolomics, acute myeloid leukemia, epigenetics, Warburg effect, redox, redox ratios, mitochondria, mitochondrial uncoupling

## Abstract

**Simple Summary:**

Myelodysplastic syndromes (MDS) encompass a very heterogeneous group of clonal hematopoietic stem cell differentiation disorders with malignant potential, an elusive pathobiology, and a poor prognosis. Given that the bioenergetic profile of the hematopoietic precursors is central to their effective differentiation, we investigated the metabolic status of a human differentiating MDS bone marrow-derived myeloid lineage. Our findings suggest that a perturbed metabolism underlies the syndrome’s pathogenesis and also determines the disease severity. We also propose that these bioenergetic alterations are essentially featured in and indeed drive the process of leukemic transformation. Our data not only offer novel insight into the elusive MDS pathophysiology, but also change our viewpoint on MDS-related acute myeloid leukemia biology.

**Abstract:**

Myelodysplastic syndromes (MDS) encompass a very heterogeneous group of clonal hematopoietic stem cell differentiation disorders with malignant potential and an elusive pathobiology. Given the central role of metabolism in effective differentiation, we performed an untargeted metabolomic analysis of differentiating myeloid lineage cells from MDS bone marrow aspirates that exhibited <5% (G1) or ≥5% (G2) blasts, in order to delineate its role in MDS severity and malignant potential. Bone marrow aspirates were collected from 14 previously untreated MDS patients (G1, *n* = 10 and G2, *n* = 4) and age matched controls (*n* = 5). Following myeloid lineage cell isolation, untargeted mass spectrometry-based metabolomics analysis was performed. Data were processed and analyzed using Metabokit. Enrichment analysis was performed using Metaboanalyst v4 employing pathway-associated metabolite sets. We established a bioenergetic profile coordinated by the Warburg phenomenon in both groups, but with a massively different outcome that mainly depended upon its group mitochondrial function and redox state. G1 cells are overwhelmed by glycolytic intermediate accumulation due to failing mitochondria, while the functional electron transport chain and improved redox in G2 compensate for Warburg disruption. Both metabolomes reveal the production and abundance of epigenetic modifiers. G1 and G2 metabolomes differ and eventually determine the MDS clinical phenotype, as well as the potential for malignant transformation.

## 1. Introduction

Every aspect of the cellular life cycle from quiescence to terminal differentiation and regulated cell death to cancerous transformation is defined by a continuous interplay between the genome and metabolome [1,2]. Proteins arbitrate such interactions by facilitating metabolic reactions while being regulated and simultaneously regulating transcriptional and metabolic processes. Acetylation [3]; Nicotinamide Adenine Dinucleotide (NAD)-dependent deacetylases; sirtuins [4]; the oncometabolite 2-Hydroxyglutarate (2HG) [5], which is notorious for its inhibitory effects upon dioxygenases; and the global methyl-group donor S-adenosyl-methionine (SAM) [6] are only a few of the countless interconnections that exist between metabolism and gene expression [1,7]. By manipulating the epigenetic modifiers, malignant cells rapidly adjust to their altering microenvironment, and their adaptation is translated into their metastatic potential [2,8].

In de novo occurring acute myeloid leukemia (AML), aberrations in transcription-related genes cause deregulation of the cell cycle and lead to leukemic transformation [9]. However, when AML develops on myelodysplastic grounds, no specific mutations have been identified [10]. Furthermore, the disease has a much worse prognosis, being mostly chemo-resistant [11]. We therefore investigated the sequence of events that transform healthy hematopoietic precursors to those presenting initially dysplastic, yet eventually blastic, offspring. The aberrant expression of Hypoxia Inducible Factor 1 (HIF1) in all differentiating myeloid lineage cells, even neutrophils and bands (I.E.S. and K.K. unpublished data, 2020) [12], indicated that metabolism may indeed play a crucial role in myelodysplastic syndromes (MDS) and MDS-related AML pathogenesis [13]. HIF1-induced metabolic reprogramming consists of a finely tuned network of extramitochondrial catabolism of glucose, despite adequate oxygen perfusion (the Warburg effect), coupled with an adequate mitochondrial function to reinstate redox balance [14]. The mitochondrial function can be broken down into two main and tightly interwoven complex biochemical processes: the Tricarboxylic Acid Cycle (TCA) and the accompanying OXidative PHOSphorylation (OXPHOS). The TCA cycle involves the cyclic oxidation of acetyl-CoA, resulting in the production of several intermediates (anabolic reactions), along with the major reducing equivalents NADH and FADH2, with both functioning as electron donors during OXPHOS [15,16]. OXPHOS is the process in which ATP is formed as a result of electron transfer from NADH or FADH2 to O2 by a series of electron carriers, also known as the mitochondrial electron transport chain (ETC) [16].

We reached the intriguing conclusion that in the MDS bone marrow (BM) microenvironment, metabolic dysregulation probably occurs and most likely majorly affects hematopoietic precursor differentiation [17]. Their resultant death is described as the ineffective erythropoiesis of MDS. The ongoing metabolic stress eventually shapes cell life, adjusting it to a new environment in a unique natural selection model. Many cells die in the process, but those which survive transform into leukemic blasts and further establish a high BM blast count MDS profile. The blasts shaped through such a strenuous process possess impressive adaptive capabilities that are largely reflected in the chemo-resistant nature of MDS-related AML.

In this study, we present the theory outlined above that was shaped through a metabolomic analysis of differentiating myeloid lineage cells from MDS BM aspirates. Differentiating myeloid lineage cells isolated from MDS BM aspirates containing <5% blasts (Group 1, G1) were compared to their counterparts isolated from MDS BM aspirates containing ≥5% blasts (Group 2, G2). G1 and G2 were also compared to differentiating myeloids from control BM aspirates.

## 2. Results

**Figure 1:**
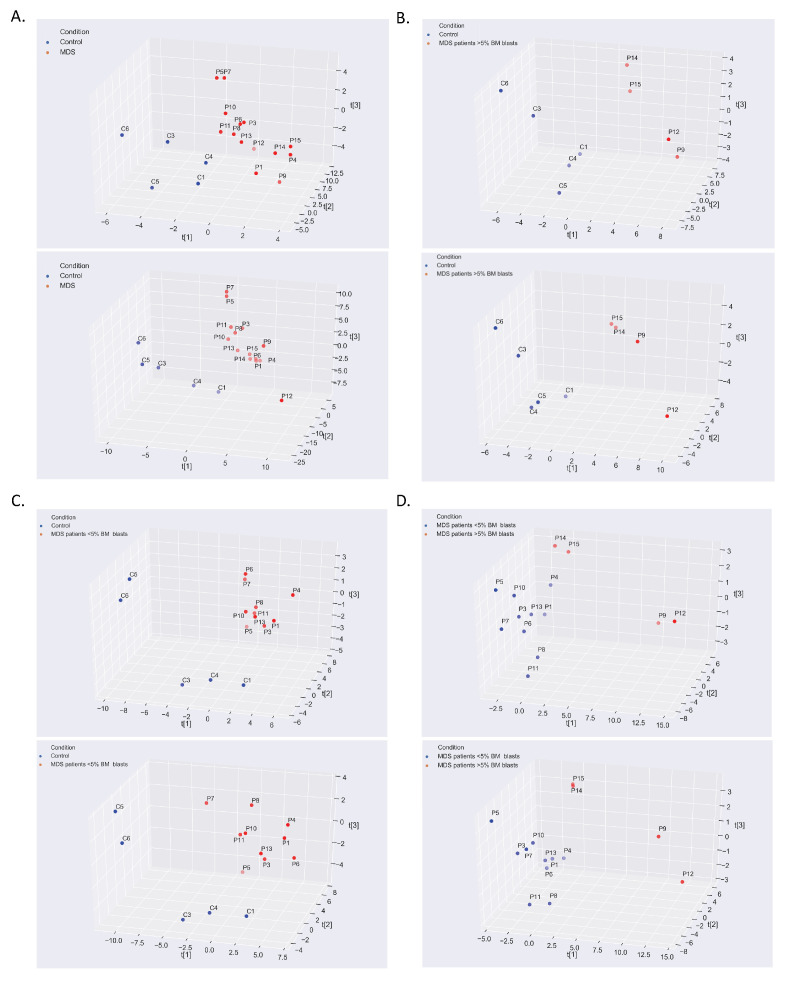
Myelodysplastic syndrome (MDS) metabotypes indicate distinct clusters for patients (high- and low-blast count MDS) and non-MDS individuals. Log2fold calculation and partial least squares (PLS) analysis were performed for (**A**) MDS vs. control metabolome, (**B**) G2 vs. control metabolome, (**C**) G1 vs. control metabolome, and (**D**) G1 vs. G2 metabolome. Upper panels correspond to negative ion mode datasets, whereas lower panels represent positive ion mode datasets (positive and negative ion modes were employed during liquid chromatography mass spectrometry analysis for metabolite characterization). G1: Differentiating myeloid lineage cells isolated from MDS bone marrow (BM) aspirates containing <5% blasts. G2: Differentiating myeloid lineage cells isolated from MDS BM aspirates containing ≥5% blasts.

The bioenergetic status of isolated BM myeloid lineage cells from MDS patients with <5% (*n* = 10; 1 MDS-SLD, 8 MDS-MLD, 1 MDS-del5q, Group 1 (G1)) and >5% BM blasts (*n* = 4; 2 MDS-EB1, 2 MDS-EB2, Group 2 (G2)) were compared to those of controls (*n* = 5), as well as with one another. MDS metabotypes indicate distinct clusters for patients (high- and low-BM blast count MDS) and non-MDS individuals, establishing the adequacy and normality of the sample selection process (Figure 1).

### 2.1. The Warburg Effect and Its Branches in the MDS Metabolome

**Figure 2:**
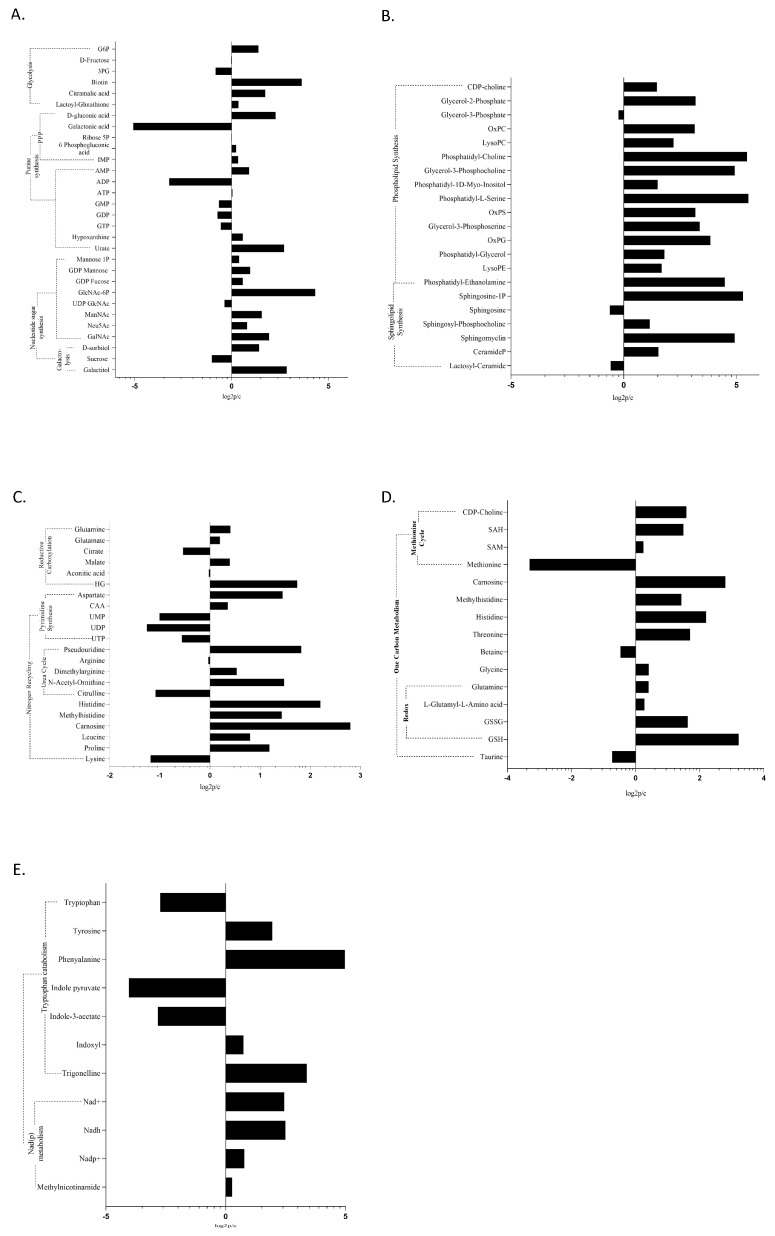
Modulation of metabolites in MDS myeloid cells compared to controls per metabolic pathway: (**A**) The Warburg effect by means of each immediate branching pathway; (**B**) sphingolipid and phospholipid metabolism; (**C**) nitrogen recycling and reductive carboxylation; (**D**) One Carbon transfer metabolism; and (**E**) nicotinate and nicotinamide metabolism. Data were processed using MetaboKit [20]. A threshold of a minimum of three samples expressing a given metabolite was set against data sparsity. Missing data were annotated with a small value (1). Data tables were scaled by data centering and setting the unit variance. Both datasets were merged in a unique data table by taking into account the maximum absolute log2fold values when a metabolite was found in both datasets. Data represent log2fold change, *p* < 0.05. G6P: Glucose-6-Phosphate; 3-PG: 3-Phosphoglycerate; 6GP acid: 6-phosphogluconic acid; IMP: inosine monophosphate; AMP: adenosine monophosphate; ATP: adenosine triphosphate; GMP: guanosine monophosphate; GDP: guanosine diphosphate; GTP: guanosine triphosphate; GlcNAc-6P: N-acetylglucosamine-6-Phosphate; ManNac: N-acetyl-D-mannosamine; Neu5Ac: 5-acetyl-neuraminate; GalNAc: N-acetyl-galactosamine; SAH: S-adenosyl-homocysteine; SAM: S-adenosyl-methionine; GSSG: glutathione disulfide; GSH: glutathione; oxPC: oxidized phosphatidyl-choline; oxPS: oxidized phosphatidyl-serine; oxPG: oxidized phosphatidyl-glycerol; PE: phosphatidyl-ethanolamine; HG: hydroxyglutarate; CAA: carbamoyl-aspartic acid; UMP: uridine monophosphate; UDP: uridine diphosphate; and UTP: uridine triphosphate.

**Figure 3:**
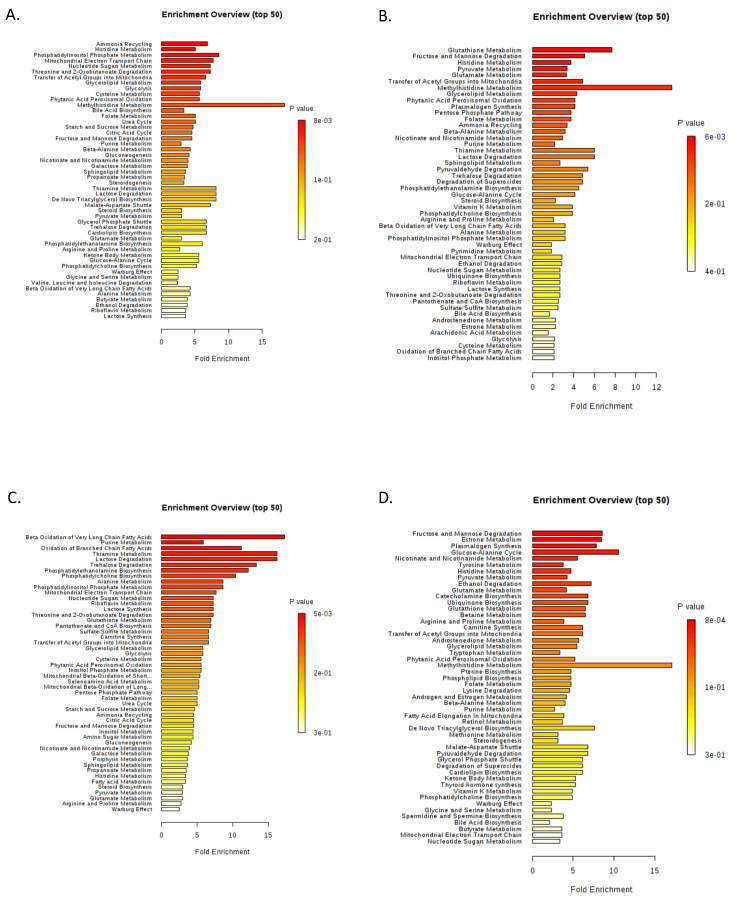
Excessive metabolic dysregulation is present in myeloid cells of MDS patients. Metabolite set enrichment analysis (MSEA) was carried out by MetaboAnalyst (v4.0). Enriched pathways were ranked by significance (see color scale) calculated by the MetPa method implemented in MetaboAnalyst (v4.0). (**A**) MDS vs. control metabolome, (**B**) G2 vs. control metabolome, (**C**) G1 vs. control metabolome, and (**D**) G1 vs. G2 metabolome. G1: Differentiating myeloid lineage cells isolated from MDS BM aspirates containing <5% blasts. G2: Differentiating myeloid lineage cells isolated from MDS BM aspirates containing ≥5% blasts.

Given the aberrant HIF1 activity in MDS, the Warburg effect, which describes the complete anaerobic oxidation of the glucose carbonic backbone, despite adequate oxygen perfusion [14], occurs in both MDS groups (G1 and G2). When comparing the MDS metabolome to that of control cells, Glucose-6-Phosphate (upper glycolysis) was found to be significantly increased (Figure 2A), while Glycerol-3-Phosphate was also upregulated in MDS containing <5% blasts vs. control cells (Figure S1A). However, as MDS differentiating cells perform Warburg glycolysis, we failed to establish significant differences between G1 and G2 groups regarding key Warburg metabolites. Furthermore, the major glycolysis branching pathway—the Pentose Phosphate Pathway (PPP) [14,18]—was significantly upregulated in the MDS group, as shown by an increase of PPP metabolites (6-Phosphogluconic, Gluconic acid), Inosine MonoPhosphate (IMP), and pyrimidine nucleotides, when compared to control cells (Figure 2A). One Carbon transfer (1C) cycle, representing another branch of upper glycolysis [18], was also significantly upregulated in the MDS metabolome; to name a few, SAM, S-Adenosyl Homocysteine (SAH), methionine, glycine, Cytidine DiPhosphate(CDP)-choline, and histidine metabolism stand out (Figure 2D). Branching from upper glycolysis, the pathway of de novo phospholipid synthesis (Cytidine 2′,3′-cyclic monophosphoric acid (CMP), CDP-choline) was deregulated in MDS, particularly in the G2 group (Figure 2B and Figure 3).

To assess the redox state of G1 and G2 groups, we determined key metabolic ratios (Table 1). This is a common practice when exploring the redox potential and bioenergetics to ensure maximum data reliability, instead of single metabolite calculations. Metabolic perturbations of interest, herein, were revealed upon data comparisons juxtaposing individual ratios per case. Engagement in Warburg glycolysis was established and further supported by a markedly increased ADP/ATP ratio, as well as a decreased pyruvate/lactate ratio, in both G1 and G2 vs. control sample comparisons (Table 1). Failure to establish a significant difference in lactate levels is probably due to the shunting of this metabolite outside the cell [19]. We established Warburg glycolysis in both G1 and G2 groups (Figure 2 and Figure 3). Unlike glycolysis and its major branching pathways PPP and 1C, every other aspect of cellular metabolism differed greatly with MDS severity.

### 2.2. The MDS Metabolome Is Dominated by Fat, While the BM Blast Count Delineates the Lipid Origin and Degradation Pathway

The most prominent feature in the MDS metabolome is lipids. Almost all metabolites that were deregulated enough to survive our statistical strictness were associated with fatty acid (and in general, lipid) metabolism (Figure 3). To be soluble in the predominantly aquatic cytoplasm, lipids are enzymatically conjugated with a hydrophilic phosphoric head, forming phospholipids. Phospholipids, including Phosphoethanolamines, Phosphatidylethanolamines (PE), Phosphatidylserines (PS), Phosphatidylinositols (PI), Phosphatidylcholines (PC), and Phosphatidylglycerol (PG), accompanied by medium, long, very long, and branched chain fatty acids were extremely upregulated in the MDS metabolome (Figure 2B). Notwithstanding, the origin and the number and type of lipids differed greatly between G1, G2, and control groups. Phospholipid upregulation became apparent in the G2 metabolome (vs. control). G1 exhibited pronounced lipid deregulation (PC, PE, monoeladin, Very Long Chain Fatty Acids (VLCFAs), and Branched Chain Fatty Acids (BCFAs)) when compared to G2 or control samples. Medium and long chain Fatty Acids (FAs) were more prominent in the G2 metabolome than that of G1.

VLCFAs/BCFAs oxidation was found to be among the most deregulated pathways when the G1 metabolome was compared to that of control samples (Figure 2C), while a significant upregulation of carnitines (palmitoyl-, oleoyl-, and propionyl-carnitine) was obtained when the G2 metabolome was compared to either control (Figure 3B) or G1 samples (Figure 3D). Βeta-oxidation of long chain FAs, as well as the transfer of Acyl-groups, with both involving functional mitochondria, were also observed in the G2 metabolome [21]. Such findings imply that beta oxidation also occurs in G1 cells, but most likely outside mitochondria inside peroxisomes, as VLCFAs and BCFAs preferentially undergo peroxisomal degradation [21,22]. Of note, carnitines, including palmitoyl-carnitine, were uniformly downregulated in G1 (Figure 3C).

The origins of FAs in MDS also varied with BM blast counts; G1 metabolomic analysis suggests an increased autophagic consumption of internal organelles metabolically denoted by markedly increased di- and tri-peptides, as well as the upregulation of ceramides (sphingomyelin (SM), Ceramide-P). Tables S2–S9 summarize the lipids assigned a Variable Importance in Projection (VIP) score greater than or equal to 1 for G1, G2, and control groups (negative- and positive-ion modes upon liquid chromatography mass spectrometry analyses). G1 and G2 exhibited increased citrate levels, indicating ongoing de novo lipid synthesis, which, at least in the G2 group, is further supported by the NADP/NADPH ratio [23] (Table 1).

### 2.3. The Optical Ratio NADH/FADH2, and the Redox Ratios NAD/NADH, GSH/GSSG, and NADP/NADPH

While, in previously published studies, a lower mitochondrial mass was found in MDS differentiating cells—to a great extent depending on uncontrolled mitophagy—the mitochondrial function has never been studied (I.E.S. and K.K. unpublished data, 2020). From a metabolic point of view, in the G2 group, mitochondrial adequacy was established through a reduced NAD/NADH ratio (when compared to the control group) in the presence of a functional TCA cycle (Table 1). The increase of the optical redox ratio of NADH/FADH2 in the G2 metabolome suggests an atypical mechanism of their mitochondrial function, specifically controlled partial uncoupling of the TCA from the ETC [24] (Figure 2 and Figure S1). The uncoupling was further supported by the massively upregulated ADP/ATP ratio detected when the G2 group was compared to the control group [24]. Electrons leaking from the ETC under these conditions form reactive oxygen species (ROS), hydrogen peroxide (H_2_O_2_), and superoxide [24,25]. A ROS-inducing metabolomic signature became evident in G2 vs. control samples, while the most impressive aspect of the G2 metabolome was the improved redox state these cells demonstrated, based on the increased reduced glutathione (GSH)/oxidized glutathione (GSSG) ratio and increased NADP and FAD levels in all comparisons. All majorly upregulated pathways in the G2 group (vs. control samples) contributed to an improved redox state through glutathione synthesis (GSH, GSSG, L-.gamma.-Glutamyl-L-glutamic Acid), nicotinate and nicotinamide metabolism (NAD, NADP, methyl nicotinamide, trigonelline, L-aspartate—Figure 2E), and ubiquinone synthesis (Figure S1B). The G1 group (vs. control samples) presented the opposite ratios: a defective redox state decreased the NADH/FADH2 ratio and as will be discussed further, induced a defective TCA cycle (Table 1).

### 2.4. Glutamine Metabolism, Nitrogen Detoxification, and the TCA in MDS

Glutamine is the major contributor of organic carbons fueling the TCA cycle in the absence of adequate influx from glycolysis [26]. Glutamine and glutamic acid were upregulated in the G1 vs. control (Figure 3C) and also in the G2 vs. G1 comparisons (Figure 3D).

In the G2 group, the reduced NAD/NADH ratio compared to control samples and FAD/FADH2 ratio compared to the G1 metabolome confirm the TCA cycle function [27] (Table 1). Additionally, the G2 group presented an increased FAD/FADH2 ratio when compared to the control one, most likely due to the aforementioned uncoupling occurring in G2 mitochondria. Conversely, in the G1 group, the TCA cycle flow seems disturbed given the high NAD/NADH, extremely high aKG/citrate, and high FAD/FADH2 ratios. Although reductive carboxylation was confirmed by the reduced aKG/citrate and increased aKG/succinate ratios in both MDS groups, the oxidative metabolism of glutamine (TCA cycle) was only present in the G2 metabolome, possibly to normalize the NADP/NADPH ratio via the regulation of NAD/NADH. The G2 metabolome also presented a higher NADP/NADPH ratio compared to G1, which further establishes the upregulated glutamine reduction in G2.

The detoxification of glutamine-derived nitrogen is confirmed via increased aspartate, urate, carbamoyl-aspartic acid, and asparagine, as well as purine and pyrimidine diphosphates [26,28]. Overall, purine and pyrimidine synthesis were the most affected pathways in the MDS metabolome (Figure 2A, Figure 3 and Figure S1). Nitrogen from glutamine was also shunted toward histidine metabolism, which is an upregulated pathway in MDS, especially in the G1 group. When comparing the MDS metabolome to that of control samples, ergothioneine, carnosine, methyl-histidine, and methyl-histamine were among the most upregulated metabolites (Figure 2).

Furthermore, glutamine metabolism is tightly bound to GSH synthesis. Both GSH and GSSG were upregulated in the MDS metabolome, yet only that of G2 was compatible with survival of the GSH/GSSG ratio, as stated above.

### 2.5. Epigenetic Modifiers Are a Distinctive Feature of MDS

MDS metabolism culminates in the reprogramming of epigenetic modifiers. SAM, which is the major intracellular methyl-donor, comes from 1C transfer and was found to be uniformly upregulated in the MDS metabolome; surprisingly more so in the G1 than G2 group (Figure 2D). We have already stated the altered usage and kinetics of acetyl-groups, mostly in the form of altered TCA cycle flow, particularly when the G2 metabolome is considered. As lysine is a major protein acetylation site, marked acetyl-lysine upregulation in the MDS vs. control metabolome indicates altered protein acetylation [3]. Furthermore, the altered NAD/NADH ratio also radically affects sirtuins (deacetylators). The ADP/ATP and GDP/GTP ratios, as revealed by MDS vs. control comparisons, may govern the enzymatic activity of epigenetic modifiers [29]. Protein glutathionylation is also thought to be affected in the MDS state given the altered levels of GSH, GSSG, and their GSH/GSSG ratio [30]. This intertwined metabolome–genome interaction reaches a crescendo with the oncometabolite 2HG. This is a byproduct of reductive glutamine carboxylation that was found to be largely upregulated in the MDS group (G2 vs. control group). The larger the 2HG area under the curve (AUC), the higher the percentage (%) of blasts in the bone marrow and thus, the higher the MDS risk.

## 3. Discussion

Cell genome and metabolome shed light on cellular function and fate, being tightly interwoven in a dynamically balanced inter-relationship [1]. Upon failing to establish a distinctive genomic profile driving the MDS phenotype and the enormous variability of genomic aberrations defining the MDS genome [10], our research focused on cell metabolic profiling. Our assumption was further supported by Hayashi et al. [31], who identified that HIF1 is highly and essentially deregulated in MDS pathogenesis, a transcription factor and master regulator of stemness metabolism, greatly dysregulated, and pathogenetically connected to the MDS phenotype [32]. Furthermore, given that disease behavior varies greatly with the percentage of BM blasts, we also hypothesized that disease severity may also be affected by the metabolic profile of the dysplastic clones, which, as proven by our analysis and depicted in Figure 1, presents high variability and disparity in comparison to the control group.

MDS are considered diseases of the Hematopoietic Stem Cell (HSC) and thus, clonal disorders [33]. It is important to mention that metabolic deregulation in the MDS BM is not confined to transformed leukemic/blastic clones, but affects every dysplastic clone. The persistence of metabolic deregulation in differentiated metamyelocytes, bands, and even neutrophils proves the clonal nature of the disorders. We believe that dysplastic cells residing under the pressure of their deregulated metabolism transform into leukemic blasts to survive—an assumption which we have yet to prove.

Differentiated cells from G2 present a viable reprogramming of their metabolism towards redox of increased ROS produced by partial uncoupling of their mitochondrial ETC to maintain mitochondrial viability when consuming, through beta oxidation, high-energy FAs [21,34]. This metabolic reprogramming established by their increased GSH/GSSG and NADH/FADH2 ratios also entails a functional TCA cycle, confirmed by reduced (vs. controls) and increased (vs. G1) NAD/NADH and FAD/FADH2 ratios, in order to retain control of the essentially active, as it will stated later, Warburg phenomenon [27,34]. Of note, Warburg glycolysis produces NADH decreasing the NAD/NADH ratio and the partially coupled ETC acts to compensate for this [14,35,36]. Glycolysis, the TCA cycle, and ETC kind of self-regulate their activity in the G2 group [36]. Aerobic glycolysis assures that glucose fluxed into the cell will not be burnt through acetyl-CoA oxidation; on the contrary, it will be used through its branching anabolic pathways, which are PPP, the 1C cycle, and phospholipid synthesis [18,36]. Mitochondrial uncoupling combined with the shunting of upper glycolysis to its branches markedly increases the ADP/ATP ratio, maintaining the Warburg phenomenon. PPP maintains the reduced NADP/NADPH ratio available for either GSSG reduction or glutamine reductive carboxylation to refeed the TCA cycle. 1C transfer provides the building blocks for nucleotides and along with the methionine cycle and PPP, is a pathway of glutamine nitrogen detoxification [26,37]. Glutamine is also used for GSH production [37], while MDS metabolism results in the vast upregulation of epigenetic modifiers. SAM, 2HG, NAD, and even GSH assure close bidirectional regulation between the metabolome and genome [8]. Finally, phospholipids, produced in masses upon either autophagy or de novo from upper glycolysis, act as secondary intra- and inter-cellular messengers allowing for the adaptation of the cell to its environment by altering the environment itself [38,39]. In fact, phospholipid deregulation is a prominent feature in the vast majority of malignancies [39]. In 2018, Stevens et al. proved that in the BM of MDS patients with >5% blasts, a distinct clonal population of CD34+/CD123+ stem-like cells with altered bioenergetics dominated by an improved redox potential and nitrogen recycling resides. These cells, which also present with upregulated protein synthesis, are thought of as the precursors of an expanding leukemic clone in these patients [40]. Interestingly, these cells presented with no increased mitotic activity, establishing the theory that leukemic stem cells (LSCs) are in fact a distinctive population of HSCs in relative quiescence. Our data support the findings of Stevens et al. and also suggest that even in the differentiating myeloids of the G2 patients, this metabolic imprint is evident. Such a metabolic profile, closely resembling that of malignant cells [41], consists of a balance between several, initially thought of as emulous, metabolic pathways and is the result of a strenuous process of trial and error, with error meaning the cellular death observed in G1.

**Figure 4:**
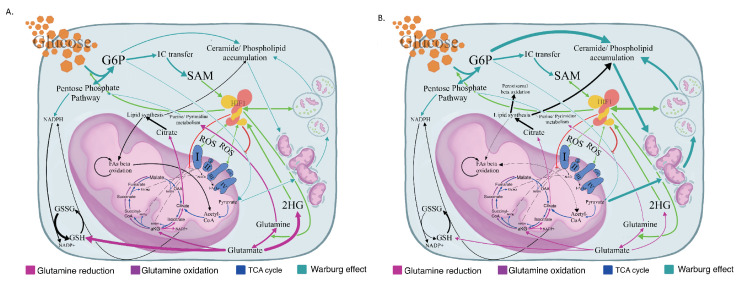
Schematic presentation of dysregulated metabolism of G1 and G2 MDS myeloid lineages. (**A**) Schematic presentation of the proposed metabolic profile of the G1 MDS differentiating myeloid precursors with predominant glycolysis, marked ceramide and phospholipid accumulation, and blunted mitochondrial function in the field of aberrant HIF1 stabilization. Line widths reflect the grade of pathway upregulation compared to controls. (**B**) Schematic presentation of the proposed metabolic profile of the G2 MDS differentiating myeloid precursors with abundant glycolysis when compared to controls, yet with an evidently improved redox status and functional mitochondrial TCA through anaplerotic reductive glutamine carboxylation. Line width reflects the extent of pathway upregulation compared to controls. SAM: S-Adenosyl-Methionine; 2-HG: 2-HydroxyGlutarate; OAA: oxaloacetate; a-Ketoglutarate: a-KG; TCA: Tricarboxylic Acid Cycle; HIF-1: Hypoxia Inducible Factor-1; Acetyl-CoA: Acetyl-Coenzyme A; and SuccinylCoA: Succinyl-Coenzyme A. Green arrows indicate enzyme upregulation by HIF-1, while the red line shows enzyme inhibition. G1: Differentiating myeloid lineage cells isolated from MDS BM aspirates containing <5% blasts. G2: Differentiating myeloid lineage cells isolated from MDS BM aspirates containing ≥5% blasts.

Indeed, differentiating cells (G1 group) presented an increased death rate when cultured in vitro (I.E.S. and K.K. unpublished data, 2020) [12]. In these cells, mitophagy is featured as the most prominent metabolic feature, probably due to dysfunctional mitochondria (I.E.S. and K.K. unpublished data, 2020) [12]. A failing TCA cycle, namely an increased NAD/NADH and FAD/FADH2 ratio with markedly increased ADP/ATP levels, leads to FA accumulation due to the failure of effective adequate beta oxidation [34,36]. The uncontrolled NAD/NADH ratio stimulates upper glycolysis in a turbo mode, which engages the cell in never-ending deadly metabolism [42]. The enormous abundance of upper glycolytic intermediates is relieved through phospholipid and ceramide synthesis [36]. FAs, mostly phospholipid and ceramide accumulation, interrupt the mitochondrial membrane lipidome. Phospholipids and ceramides are the cellular membrane building blocks. Mitochondrial membrane disorganization equals ETC dysfunction and thus, ROS leakage [38,43]. Eventually, damaged mitochondria are targeted for autophagic consumption, further increasing phospholipid release and incapacitating mitochondrial function fulfilling this deadly cycle [43]. Increased ROS levels and hence, increased oxidative stress, are featured in our analysis as a decreased GSH/GSSG ratio, despite the increased GSH production. Of note, though, the seemingly improved GSH/GSSG ratio in the G1 vs. G2 comparison is most likely the result of massively upregulated glutamine metabolism. The latter does not result in an improved redox state, as proved by the G1 vs. control and G2 vs. control comparisons. Instead, increased glutathionylation and the functional impairment of mitochondrial proteins became apparent in G1 [30,44]. Furthermore, the failure of mitochondria to oxidize FAs leads to their accumulation and peroxisomal degradation. Peroxisomal biogenesis is stimulated upon the increased needs of peroxisomes and the endoplasmic reticulum is sequentially consumed in the process of autophagosome formation and peroxisome-genesis, eventually conferring death of the cell [13,45,46]. Figure 4 summarizes the metabolomic profile of both G1 (Figure 4A) and G2 (Figure 4B).

Due to the scarcity of such samples in our patient pool, our cohort did not include clinical cases of MDS with ring sideroblasts (RS). Notwithstanding, we addressed this challenge via in silico means. Current knowledge and datasets were reviewed in an effort to compare the bioenergetic state of MDS-RS, representing this distinct relatively benign MDS subgroup, with that of our G1 myeloid lineage. The vast majority of MDS-RS cases reported are dominated by *SF3B1* mutations [47]. While *SF3B1* mutations drive the formation of RS in the erythroid lineage of the dysplastic clone, a veil of mystery surrounds their effects on the myeloid sub-clone [48]. From a metabolomic perspective, *SF3B1* mutations have been shown to cause profound defects in the ETC and thus in mitochondrial respiration due to the impairment of Complex III assembly [49] in the differentiating myeloid lineage of MDS-RS [48].

## 4. Materials and Methods

### 4.1. Sample Collection and Preparation

Isolated BM myeloid cells (high-density layer) from MDS patients with <5% (*n* = 10; 1 MDS-SLD, 8 MDS-MLD, 1 MDS-del5q, Group 1 (G1)) and >5% BM blasts (*n* = 4; 2 MDS-EB1, 2 MDS-EB1, Group 2 (G2)) and aged matched controls (*n* = 5) were subjected to untargeted mass spectrometry-based metabolomics analysis (Table S1). Two different metabolite extraction protocols were applied (1° extraction: 50% MeOH, 30% ACN, 20% H_2_O; 2° extraction: 40% MeOH, 40% EtOH, 20% H_2_O). The first extraction procedure was chosen for the best metabolite yield. In total, 50 µL of samples was used for metabolite extraction by adding 750 µL of extraction buffer and shaking the mixture at 4 °C for 15 min. Samples were then centrifuged at 14,000 *g* for 10 min and supernatants were collected for LC-MS/MS analysis using the UPLC 1290 system (Agilent Technologies, Santa Clara, CA USA) coupled to a TripleTOF 5600+ mass spectrometer (SCIEX) equipped with SWATH acquisition, SelexION technology, and an electrospray ionization source (ESI). Both G1 and G2 samples along with control ones were examined under optical microscopy to assure equal representation of every myeloid differentiation stage in all samples. To exclude the possibility of an artificial myeloid lineage disturbed metabolome due to contamination of the high-density layer of G2 samples from blastic cells, we performed a control BM ficoll bilayer isolation protocol on a BM aspirate of a patient with MDS-EB2 and assessed the isolated layer, namely the myeloid layer, with flow cytometry, after staining for appropriate antigens. The flow cytometry acquisition was performed on a FACSCanto II flow cytometer. Flow cytometry analysis was performed using FlowJo software. A purity of >95% mature myeloid lineage with less than 0.1% CD34+ blastic contamination was established. Flow cytometric tables from both the BM aspirate and the isolated layer are shown in Figure S2.

### 4.2. Chromatography

UPLC separation was performed on metabolite extracts using a pZIC-HILIC column (5 µm, 2.1 mm × 150 mm) operating at 45 °C by directly injecting 10 µL of samples. The flow rate was 0.2 mL/min with mobile phase A (acetonitrile) and mobile phase B (ammonium carbonate 20 mM + 0.1% ammonium hydroxide). The gradient, in both positive and negative mode, was from 20% to 80% B in 15 min.

### 4.3. Mass Spectrometry

The TripleTOF 5600+ system was used for data acquisition, over a mass range of 75–1000 m/z. Automated calibration was performed using an external calibrant delivery system (CDS), which infuses APCI positive or negative calibration solution every five samples. To monitor the instrument performance over time, quality control samples (QC) were prepared as a mix of each sample and analyzed every five samples. A TOF MS survey scan experiment with an IDA set to monitor the eight most intense candidate ions was performed (accumulation time of 150 msec in TOF-MS and 50 msec in the IDA experiment), with a collision energy of 35 eV and a collision energy spread of 10 eV, a declustering potential of 80 V, a source temperature (TEM) of 500 °C, and IonSpray Voltage Floating (ISVF) of 5500 V (positive polarity) or 4500 V (negative polarity) in high-sensitivity mode.

### 4.4. Data Processing

Data were processed and analyzed using MetaboKit [20] (https://github.com/MetaboKit/MetaboKit). Patient data were separated into two cohorts, depending on the severity of MDS disease (G1, *n* = 10 and G2, *n* = 4). All groups were cross-compared, resulting in a total of three different analyses: non-MDS vs. G1; non-MDS vs. G2; and G1 vs. G2. These comparisons were designed first to obtain insights into the MDS metabolome at different disease stages and then, identify key metabolites differentiating G1 and G2.

Data processing and analysis for each experiment were performed after employing both positive and negative ion modes during liquid chromatography mass spectrometry analysis. Metabolites present in more than 33% of the samples were included in the analyses. After removing uninformative features, the resulting number of metabolites was decreased drastically to ~¼. For those metabolites passing the criteria, empty values were annotated with a small value (1). Data centering and unit variance scaling were performed. Log2fold calculation, PCA, and PLS analyses were performed for every comparative analysis (both negative and positive ion modes). PLS VIP (variable importance in projection) values were determined. Only metabolites with a log2fold ≥ 2 were selected for subsequent enrichment analysis. Enrichment analysis was performed using Metaboanalyst v4 [50] employing pathway-associated metabolite sets (SMPDB).

### 4.5. Study Approval

The study protocol is in accordance with the Declaration of Helsinki and has been approved by the ethics review board of Athens General Hospital “Laiko”. IRB protocol number: 13011. Written informed consent was obtained from each individual participating in the study.

## 5. Conclusions

In the G1 group, the Warburg effect predominates. The silencing of the mitochondrial function with increased NAD+/NADH and markedly increased ADP/ATP ratios increases cellular oxidative pressure. In fact, the GSH/GSSG and NADP+/NADPH ratios were decreased and increased accordingly, when compared to the control group, underlining the increased oxidative stress that low-risk precursors reside in. On the contrary, the G2 metabolome presented with opposite ratios. The relief of an increase NAD+/NADH ratio as we suggest via the mitochondrial TCA cycle allows for the high blast count MDS myeloid precursors to utilize aerobic glycolysis for anabolic processes. Of note, the mitochondrial function in this latter group of MDS was supported by our analysis. In fact, a moderately decreased ADP/ATP ratio permits the viable utilization of the Warburg effect. When these precursors acquire the ability to balance aerobic glycolysis and mitochondrial oxidative stress, AML transformation occurs. We hypothesize that due to an initiating event, which we have yet to identify, HSCs fail to shut down HIF1 metabolism and enter the G1 bioenergetic mode. Under the resultant continuous metabolic stress and epigenetic modifier abundance, low blast count MDS transforms to its high-risk counterpart and eventually to AML blasts. Identifying this event and meticulously ascribing the MDS metabolome will offer improved therapeutic strategies against this enigmatic entity.

## Figures and Tables

**Table 1:** Important metabolic ratios that decipher the MDS metabolome.

Ratios	G1 vs. Controls	G2 vs. Controls	G1 vs. G2
NAD+/NADH	3.76	0.97	0.41
ADP/ATP	6.82	26.01	0.95
Citrate/aKG	12.45	3.77	0.38
GSH/GSSG	0.86	3.35	3.87
NADP+/NADPH	1.21	0.25	0.21
FAD/FADH2	2.19	2.94	2.22
NADH/FADH2	1.02	1.34	0.48
Pyruvate/Lactate	0.97	0.83	2.31

G1: Differentiating myeloid lineage cells isolated from MDS BM aspirates containing <5% blasts. G2: Differentiating myeloid lineage cells isolated from MDS BM aspirates containing ≥5% blasts.

## Supplementary Materials

The following are available online at https://www.mdpi.com/2072-6694/12/12/3520/s1: Figure S1: Key metabolic pathways in MDS profiling; Figure S2: Control BM flow cytometry; Table S1: MDS patient characteristics; Table S2: MDS myeloid cell metabolome compared to that of non-MDS (control group). LC-MS analysis in negative ion mode; Table S3: MDS myeloid cell metabolome compared to that of non-MDS (control group). LC-MS analysis in positive ion mode; Table S4: G2 metabolome compared to that of non-MDS (control group). LC-MS analysis in negative ion mode; Table S5: G2 metabolome compared to that of non-MDS (control group). LC-MS analysis in positive ion mode; Table S6: G1 metabolome compared to that of non-MDS (control group) LC-MS analysis in negative ion mode; Table S7: G1 metabolome compared to that of non-MDS (control group) LC-MS analysis in positive ion mode; Table S8: G1 metabolome compared to that of G2. LC-MS analysis in negative ion mode; Table S9: G1 metabolome compared to that of G2. LC-MS analysis in positive ion mode.
