# Supplementary material for: Bioenergetic Profiling of the Differentiating Human MDS Myeloid Lineage with Low and High Bone Marrow Blast Counts
Source: Cancers (Basel). 2020 Nov 26;12(12):3520. doi: 10.3390/cancers12123520 (PMC7759906; doi:10.3390/cancers12123520)
Supplement: Supplementary file 1 [file cancers-12-03520-s001.pdf]

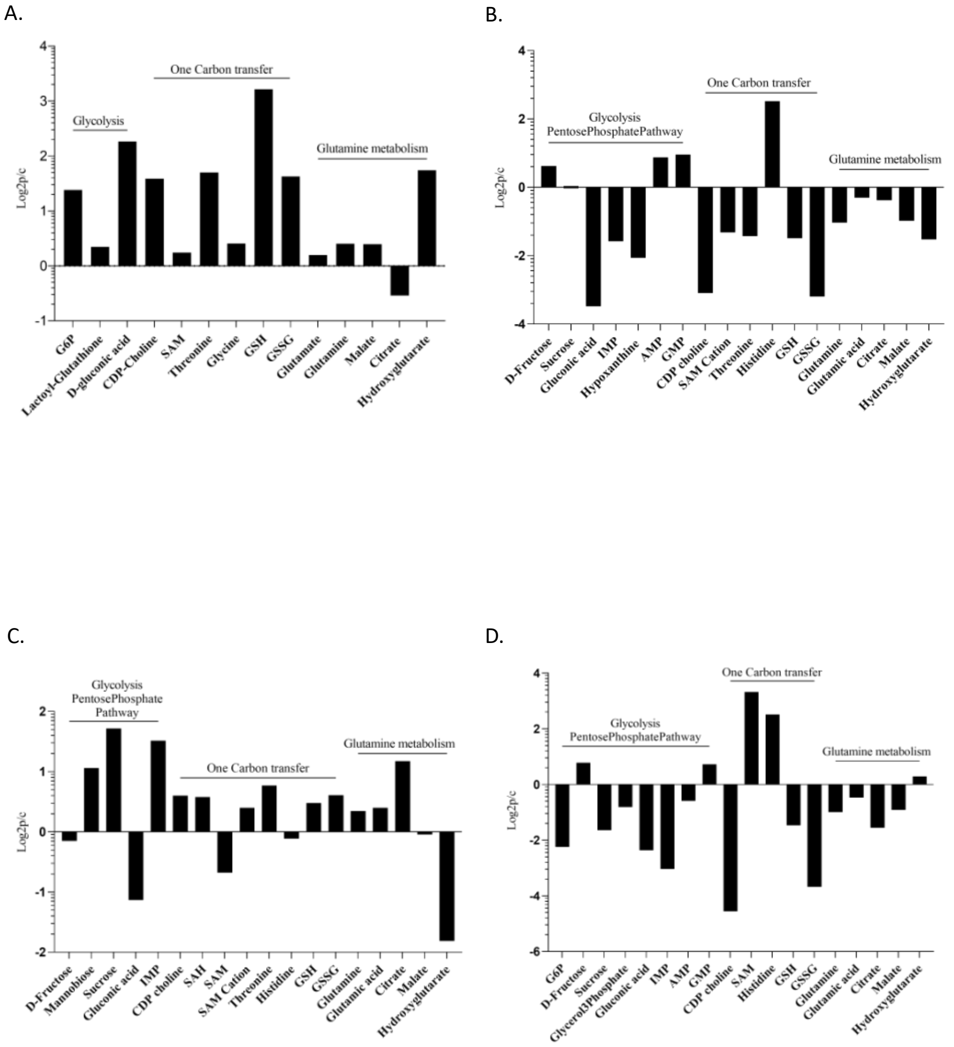

Supplementary Figure 1. Key metabolic pathways in MDS profiling. Modulation of metabolites per key metabolic pathway in (A) MDS myeloid cells compared to controls; (B) G2 vs. controls; (C) G1 vs. controls; (D) G1 vs. G2. Data represent log2fold change, $p<0,05$. G6P: Glucose-6-Phosphate, CDP-choline: cytidine-diphospho-choline, SAM: S-adenosyl-methionine, GSH: Glutathione, GSSG: Glutathione disulfide. G1: Differentiating myeloid lineage cells isolated from MDS BM aspirates containing <5% blasts, G2: Differentiating myeloid lineage cells isolated from MDS BM aspirates containing ≥5% blasts.

A.

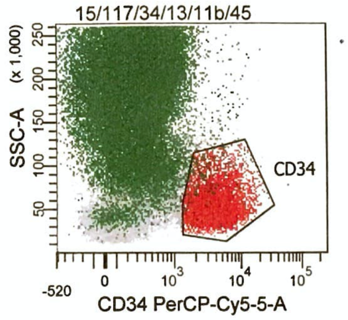

Tube: 15/117/34/13/11b/45

Population	#Events	%Parent	%Total
All Events	216,348	####	100.0
SINGLETS	162,505	75.1	75.1
NUCLEATED CELLS	133,498	82.2	61.7
CD45	75,482	56.5	34.9
CD34	6,851	9.1	3.2
CD11B	38,849	51.5	18.0

B.

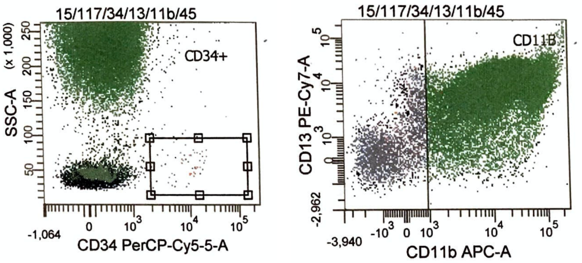

Tube: 15/117/34/13/11b/45

Population	#Events	%Parent	%Total
All Events	115,170	####	100.0
SINGLETS	94,464	82.0	82.0
NUCLEATED CELLS	85,854	90.9	74.5
CD45	82,811	96.5	71.9
CD34+	62	0.1	0.1
CD11B	79,951	96.5	69.4

Supplementary Figure 2: **Control BM flow cytometry.** Flow cytometric analysis of (A) BM aspirate from a patient with MDS-EB2 (9% blasts) and (B) isolated high density layer of the same patient after ficoll bilayer isolation protocol. The flow cytometry acquisition was performed on a FACSCanto II flow cytometer. Flow cytometry analysis was performed using FlowJo software. A purity of >95% maturing myeloid lineage with 0.1% CD34+ contamination was established.

Patient ID	Age (years)	Gender	MDS subtype	IPSS-R	Hb (g/dl)	WBC (K/µl)	Neutrophils (K/µl)	Platelets (K/µl)	BM Cytogenetics	Peripheral Blood Blasts (% of nucleated cells)†	BM Blasts (% of nucleated cells)††	BM cellularity*	Transfusion Dependence
1	81	Female	MLD	1 (very low)	11,3	4,74	2,70	139	46,XX[20]	0	2	Normocellular	No
2	83	Male	MLD	1 (very low)	13,0	3,53	1,90	142	46,XY[20]	0	2	Hypocellular	No
3	78	Male	MLD	2,5 (low)	6,6	3,25	1,25	116	46,XY[20]	0	3	Hypocellular	Yes
4	74	Female	Isolated del(5q)	2 (low)	8,5	2,77	1,67	386	46,XX,del(5q)[20]	0	1	Normocellular	
5	82	Female	MLD	1 (very low)	10,6	4,50	3,15	177	46,XX[20]	0	2	Normocellular	No
6	77	Male	MLD	1 (very low)	9,3	4.90	2,74	324	46,X,-Y[20]	2	3	Normocellular	Yes
7	80	Male	SLD	3 (low)	8,8	8,18	5,64	520	46,XY[20]	0	0	Hypercellular	Yes
8	78	Male	MLD	3 (low)	9,5	2,38	1,67	148	46,XY[20]	0	4	Normocellular	Yes
9	77	Male	EB 1	6 (high)	9,4	1,83	0,60	56	46,XY, del(5)(q11.2q33), del(20)(q11.2 ~13.1)[18]/46,XY[1]	1	8	Normocellular	
10	85	Male	MLD	3,5 (intermediate)	5,6	3.48	2,50	131	47,XY,+8[20]/46,XY[5]	0	0	Normocellular	Yes
11	63	Male	MLD	2 (low)	10,7	3,16	0,90	274	46,XY, inv(9)(p12q13)[12]	0	0	Hypocellular	No
12	79	Female	EB 2	6 (high)	9,0	4,47	2,00	103	47,XX,+8[20]	5	15	Hypercellular	No
13	90	Male	EB 1	7,5 (very high)	4,7	9,32	6,90	184	46,XY, del(7)(q21)[6]/45,XY?add(1)(p36),add(2)(q37),del(7)(q21),-20[15]	2	8	Normocellular	Yes
14	75	Male	EB 2	7 (very high)	8,6	1,90	0,50	58	47,XY,+8[23]/46,XY[2]	3	15	Hypercellular	Yes
15	81	Male	MLD	3(low)	8,0	1,78	0,50	145	47,XY,+8[23]/46,XY[2]	0	0	Hypercellular	No
16	81	Male	MLD	2(low)	8,9	2,11	1,0	120	46,XY, del(20)(q11.2)[30]	0	1	Hypercellular	No

Supplemental Table 1. MDS Patients characteristics.

Hb: hemoglobin, WBC: white blood cells † assessed by morphology in peripheral blood smears,†† assessed by morphology of bone marrow smears, *assessed in bone marrow biopsy

Lipids with VIP score greater than or equal to 1					
ID	VIP	FORMULA	COEF	FOLD CHANGE (FC)	log2(FC)
369	1.67299	C42H84NO7P	-0.0464661	0.3521	-1.506
179	1.59156	C20H30O5	-0.0193666	0.19355	-2.3692
384	1.57968	C45H82NO7P	-0.0460789	0.44171	-1.1788
454	1.57418	C47H83O13P	-0.0181425	0.58358	-0.77699
382	1.56125	C45H80NO7P	-0.0348027	0.3613	-1.4687
274	1.51046	C34H34N4O4	-0.0139848	0.1757	-2.5088
376	1.44003	C44H86NO7P	-0.0416105	NA	NA
169	1.43633	C22H43NO	-0.0148299	0.13294	-2.9112
449	1.39729	C46H76NO10P	-0.0113553	NA	NA
380	1.35439	C45H78NO7P	0.0381154	22.265	4.4767
305	1.34174	C37H75N2O6P	-0.0229592	0.66298	-0.59296
453	1.29929	C46H87NO13	-0.0314464	0.66578	-0.58689
326	1.24561	C39H80N2O6P	0.0224264	14.437	3.8517
327	1.23729	C41H76NO7P	-0.0332823	NA	NA
393	1.23415	C44H84NO8P	0.0357335	1.2376	0.30759
349	1.22073	C42H84NO7P	0.0040528	0.48929	-1.0312
183	1.20844	C21H40O4	-0.0008714	0.59655	-0.74528
383	1.20777	C42H80NO8P	-0.0117198	0.30917	-1.6935
423	1.19005	C46H84NO8P	-0.0183378	0.39158	-1.3526
389	1.1852	C42H84NO8P	-0.000982	0.49609	-1.0113
359	1.17911	C42H82NO8P	-0.0087269	0.56196	-0.83147
251	1.14855	C26H56NO6P	-0.0181191	NA	NA
203	1.11676	C24H38O4	-0.0028356	1.883	0.91304
370	1.11313	C44H82NO7P	-0.0207497	0.23794	-2.0713
357	1.09539	C42H80NO8P	0.0101928	0.76901	-0.37892
304	1.07597	C37H73N2O6P	-0.0163743	0.4492	-1.1546
208	1.06166	C23H45NO4	-0.0075633	0.48287	-1.0503
333	1.05824	C40H78NO8P	-0.008696	0.59894	-0.73952
248	1.05197	C26H54NO6P	-0.012436	0.65592	-0.60841
350	1.04853	C43H74NO7P	0.0178384	1.3289	0.41024
437	1.04825	C48H84NO7P	0.0244126	NA	NA
379	1.03833	C42H79O10P	0.0175388	4.4189	2.1437
272	1.02962	C26H54NO7P	0.0150358	7.5182	2.9104
197	1.02332	C18H38NO5P	0.00939553	4.0026	2.0009
317	1.0227	C39H79N2O6P	0.0122224	1.9648	0.97437
137	1.00846	C18H37NO2	-0.0024647	0.64887	-0.624

Supplemental Table 2. MDS myeloid cell metabolome compared to that of non-MDS (control group). LC-MS analysis in negative ion mode

Lipids with VIP score greater than or equal to 1					
ID	VIP	FORMULA	COEF	FOLD CHANGE (FC)	log2(FC)
369	1.67299	C42H84NO7P	-0.0464661	0.3521	-1.506
179	1.59156	C20H30O5	-0.0193666	0.19355	-2.3692
384	1.57968	C45H82NO7P	-0.0460789	0.44171	-1.1788
454	1.57418	C47H83O13P	-0.0181425	0.58358	-0.77699
382	1.56125	C45H80NO7P	-0.0348027	0.3613	-1.4687
274	1.51046	C34H34N4O4	-0.0139848	0.1757	-2.5088
376	1.44003	C44H86NO7P	-0.0416105	NA	NA
169	1.43633	C22H43NO	-0.0148299	0.13294	-2.9112
449	1.39729	C46H76NO10P	-0.0113553	NA	NA
380	1.35439	C45H78NO7P	0.0381154	22.265	4.4767
305	1.34174	C37H75N2O6P	-0.0229592	0.66298	-0.59296
453	1.29929	C46H87NO13	-0.0314464	0.66578	-0.58689
326	1.24561	C39H80N2O6P	0.0224264	14.437	3.8517
327	1.23729	C41H76NO7P	-0.0332823	NA	NA
393	1.23415	C44H84NO8P	0.0357335	1.2376	0.30759
349	1.22073	C42H84NO7P	0.0040528	0.48929	-1.0312
183	1.20844	C21H40O4	-0.0008714	0.59655	-0.74528
383	1.20777	C42H80NO8P	-0.0117198	0.30917	-1.6935
423	1.19005	C46H84NO8P	-0.0183378	0.39158	-1.3526
389	1.1852	C42H84NO8P	-0.000982	0.49609	-1.0113
359	1.17911	C42H82NO8P	-0.0087269	0.56196	-0.83147
251	1.14855	C26H56NO6P	-0.0181191	NA	NA
203	1.11676	C24H38O4	-0.0028356	1.883	0.91304
370	1.11313	C44H82NO7P	-0.0207497	0.23794	-2.0713
357	1.09539	C42H80NO8P	0.0101928	0.76901	-0.37892
304	1.07597	C37H73N2O6P	-0.0163743	0.4492	-1.1546
208	1.06166	C23H45NO4	-0.0075633	0.48287	-1.0503
333	1.05824	C40H78NO8P	-0.008696	0.59894	-0.73952
248	1.05197	C26H54NO6P	-0.012436	0.65592	-0.60841
350	1.04853	C43H74NO7P	0.0178384	1.3289	0.41024
437	1.04825	C48H84NO7P	0.0244126	NA	NA
379	1.03833	C42H79O10P	0.0175388	4.4189	2.1437
272	1.02962	C26H54NO7P	0.0150358	7.5182	2.9104
197	1.02332	C18H38NO5P	0.00939553	4.0026	2.0009
317	1.0227	C39H79N2O6P	0.0122224	1.9648	0.97437
137	1.00846	C18H37NO2	-0.0024647	0.64887	-0.624

Supplemental Table 3. MDS myeloid cell metabolome compared to that of non-MDS (control group). LC-MS analysis in positive ion mode

Lipids with VIP score greater than or equal to 1					
ID	VIP	FORMULA	COEF	FOLD CHANGE (FC)	log2(FC)
303	1.77137	C43H74NO7P	-0.0749743	9.8149	3.295
282	1.76826	C41H80NO7P	-0.0865403	3.5157	1.8138
323	1.58163	C43H78NO8P	0.0517995	0.20387	-2.2943
126	1.46362	C20H38O2	-0.0242533	19.455	4.2821
392	1.38602	C46H80NO10P	0.0245433	0.05835	-4.0991
306	1.37012	C43H76NO7P	-0.0484352	1.7628	0.81791
408	1.30578	C45H85O13P	-0.0121136	8.2019	3.036
232	1.28481	C27H53O12P	-0.037725	2.5861	1.3708
262	1.28455	C39H76NO7P	-0.0495741	2.0221	1.0158
393	1.27746	C46H82NO10P	0.0130141	0.16864	-2.568
251	1.25278	C37H69O8P	0.0144587	0.1335	-2.9051
342	1.2475	C45H82NO7P	0.0149183	0.35362	-1.4997
410	1.24601	C45H83O13P	-0.0221597	3.6458	1.8662
266	1.1723	C39H76NO8P	0.0102356	0.40989	-1.2867
407	1.15208	C45H83O13P	-0.0042759	2.7955	1.4831
374	1.14308	C44H76NO10P	0.00203827	0.29466	-1.7629
376	1.11657	C44H78NO10P	0.00870088	0.37545	-1.4133
287	1.09655	C41H74NO8P	-0.0035131	0.37527	-1.414
231	1.08329	C27H51O12P	-0.0121638	2.5861	1.3708
264	1.06395	C39H74NO8P	0.0137117	0.40031	-1.3208
308	1.0311	C43H78NO7P	-0.0273842	0.63197	-0.66207
142	1.02439	C22H36O2	-0.0256213	2.3473	1.231

Supplemental Table 4. G2 metabolome compared to that of non-MDS (control group). LC-MS analysis in negative ion mode

Lipids with VIP score greater than or equal to 1					
ID	VIP	FORMULA	COEF	FOLD CHANGE (FC)	log2(FC)
454	1.6029	C47H83O13P	-0.0506303	2.4627	1.3002
453	1.60134	C46H87NO13	-0.0549459	3.9139	1.9686
183	1.59573	C21H40O4	-0.0349459	42.126	5.3966
369	1.59105	C42H84NO7P	-0.0603147	110.29	6.7852
403	1.40332	C42H80NO10P	-0.0503103	3.1293	1.6458
390	1.39647	C44H82NO8P	-0.0499924	19.495	4.2851
423	1.38073	C46H84NO8P	-0.0352844	141.17	7.1413
382	1.37022	C45H80NO7P	-0.0535288	5.3179	2.4109
389	1.32988	C42H84NO8P	-0.0252421	5.9296	2.5679
330	1.30496	C41H81N2O6P	0.0173829	0.14109	-2.8253
317	1.30309	C39H79N2O6P	0.0192753	0.24687	-2.0182
418	1.28128	C42H78NO10P	-0.0268764	31.923	4.9965
325	1.248	C41H74NO7P	-0.0385346	2.8752	1.5237
394	1.21938	C44H84NO8P	-0.0370931	1803.2	10.816
5	1.13297	C2H7NO3S	-0.0294512	2.4339	1.2833
328	1.10154	C41H78NO7P	-0.0407644	4.6973	2.2318
393	1.09702	C44H84NO8P	0.0410293	0.93314	-0.099836
424	1.03125	C47H91N2O6P	0.00215556	0.42135	-1.2469
348	1.02143	C41H80NO8P	-0.0362665	1.791	0.84075
442	1.0068	C44H78NO10P	0.0100563	0.37715	-1.4068
261	0.994222	C26H54NO7P	0.0209805	0.27089	-1.8842

Supplemental Table 5. G2 metabolome compared to that of non-MDS (control group). LC-MS analysis in positive ion mode

Lipids with VIP score greater than or equal to 1					
ID	VIP	FORMULA	COEF	FOLD CHANGE (FC)	log2(FC)
332	1.66515	C42H79O11P	0.0777219	0.46266	-1.112
324	1.49286	C43H78NO8P	-0.0860096	2.6369	1.3988
307	1.38128	C43H78NO7P	-0.0740215	3.6469	1.8667
196	1.3537	C23H46NO7P	-0.0732541	1.7247	0.78637
277	1.33043	C41H78NO7P	0.0634909	0.57371	-0.8016
212	1.3139	C24H46NO9P	-0.0316119	3.9641	1.987
302	1.29242	C40H75O10P	0.0565096	0.26904	-1.8941
138	1.22993	C18H30O3S	0.0666913	0.67926	-0.55797
353	1.2288	C42H78NO10P	-0.0506572	1.6782	0.74695
215	1.21639	C24H48NO9P	-0.0380766	1.8066	0.85325
244	1.18487	C35H69O8P	-0.0534653	2.9863	1.5784
410	1.16166	C45H83O13P	-0.0371914	2.3441	1.229
210	1.15229	C24H47O9P	0.031616	1.1559	0.20903
231	1.10048	C27H51O12P	0.00879756	1.6188	0.6949
232	1.09376	C27H53O12P	-0.0278768	1.5541	0.63607
240	1.09038	C29H49O12P	-0.0304224	1.7502	0.80756
323	1.0476	C43H78NO8P	0.0318223	0.72716	-0.45966
351	1.04651	C42H76NO10P	0.0364566	0.46096	-1.1173
262	1.03788	C39H76NO7P	-0.0542978	1.478	0.5636
306	1.03455	C43H76NO7P	-0.0486245	1.6958	0.76198
304	1.0257	C40H77O10P	0.0249812	1.0893	0.12345

Supplemental Table 6. G1 metabolome compared to that of non-MDS (control group) LC-MS analysis in negative ion mode

Lipids with VIP score greater than or equal to 1					
ID	VIP	FORMULA	COEF	FOLD CHANGE (FC)	log2(FC)
317	1.60246	C39H79N2O6P	0.0586366	0.88465	-0.17683
143	1.53241	C20H32O2	0.0796232	0.51792	-0.94919
350	1.3839	C43H74NO7P	0.0439179	0.61715	-0.69632
369	1.36118	C42H84NO7P	-0.0707108	2.0419	1.0299
325	1.31432	C41H74NO7P	0.0266586	0.80537	-0.31228
402	1.22401	C42H80NO10P	0.0421728	0.85839	-0.22029
256	1.22038	C26H50NO7P	0.0310498	1.0749	0.10421
393	1.19593	C44H84NO8P	0.0567897	0.76683	-0.38302
397	1.13169	C42H78NO10P	-0.0454384	1.7492	0.80668
305	1.12928	C37H75N2O6P	-0.0368635	1.8019	0.84956
413	1.09471	C44H84NO8P	-0.0355022	1.8403	0.87996
328	1.08677	C41H78NO7P	-0.0396239	1.1825	0.24179
238	1.08642	C24H52NO6P	0.0101569	1.5859	0.6653
203	1.07666	C24H38O4	0.00048243	0.51265	-0.96394
453	1.07611	C46H87NO13	-0.043287	1.2049	0.26888
315	1.07537	C39H77N2O6P	-0.0192131	1.7233	0.78514
394	1.06748	C44H84NO8P	0.0178442	0.8164	-0.29265
382	1.0588	C45H80NO7P	-0.0564405	2.3208	1.2146
258	1.05142	C26H52NO7P	-0.0208985	1.7073	0.77169
309	1.03808	C38H77N2O6P	-0.0302047	1.6262	0.70155
454	1.01925	C47H83O13P	-0.0249137	1.5268	0.61052
358	1.01861	C42H80NO8P	-0.0363448	13.637	3.7695
244	1.0127	C24H50NO7P	-0.0277706	1.8792	0.91015

Supplemental Table 7. G1 metabolome compared to that of non-MDS (control group) LC-MS analysis in positive ion mode

Lipids with VIP score greater than or equal to 1					
ID	VIP	FORMULA	COEF	FOLD CHANGE (FC)	log2(FC)
323	1.56632	C43H78NO8P	0.151883	0.27918	-1.8408
408	1.32335	C45H85O13P	-0.0670108	7.2012	2.8482
98	1.32293	C12H26O4S	0.0921068	0.80035	-0.3213
264	1.31462	C39H74NO8P	0.00958981	0.22411	-2.1578
251	1.31229	C37H69O8P	-0.0114198	0.10257	-3.2854
332	1.30547	C42H79O11P	-0.0131707	8.9215	3.1573
282	1.29458	C41H80NO7P	-0.0899385	2.9836	1.5771
392	1.2815	C46H80NO10P	0.00796141	0.16842	-2.5699
374	1.26622	C44H76NO10P	-0.0014927	0.18398	-2.4424
300	1.24799	C41H80NO8P	0.0870158	1.2335	0.30274
393	1.24487	C46H82NO10P	0.01055	0.20086	-2.3157
137	1.24462	C18H30O3S	0.0997555	1.2662	0.34046
320	1.20227	C43H74NO8P	0.0283447	0.20561	-2.2821
258	1.17415	C39H74NO7P	-0.0945788	10.907	3.4471
389	1.16122	C46H78NO10P	0.00200301	0.23671	-2.0788
322	1.135	C43H76NO8P	-0.0078362	0.34093	-1.5525
138	1.10424	C18H30O3S	-0.110132	1.4981	0.58318
266	1.09911	C39H76NO8P	0.0230982	0.45714	-1.1293
376	1.08914	C44H78NO10P	0.0137641	0.36168	-1.4672
196	1.08141	C23H46NO7P	0.0988699	0.67187	-0.57374
303	1.06103	C43H74NO7P	-0.0762404	6.0124	2.5879
277	1.04281	C41H78NO7P	0.0622577	1.4233	0.50928
287	1.03826	C41H74NO8P	-0.0086705	0.43916	-1.1872
317	1.02767	C40H76NO10P	-0.0110579	0.3711	-1.4301
407	1.02361	C45H83O13P	-0.0363555	1.9803	0.98572
324	1.01509	C43H78NO8P	0.100518	0.6529	-0.61506
308	1.00897	C43H78NO7P	-0.0398775	0.55399	-0.85208
351	1.00442	C42H76NO10P	-0.0069644	0.30229	-1.726
423	0.997442	C49H87O13P	-0.0353915	29.927	4.9034
232	0.996333	C27H53O12P	-0.0126688	1.6684	0.73846

Supplemental Table 8. G1 metabolome compared to that of G2.
LC-MS analysis in negative ion mode

Lipids with VIP score greater than or equal to 1					
**ID**	**VIP**	**FORMULA**	**COEF**	**FOLD CHANGE (FC)**	**log2(FC)**
331	1.60324	C41H80NO7P	-0.114233	17.385	4.1198
313	1.55103	C39H74NO7P	-0.145873	22.131	4.468
359	1.48292	C42H82NO8P	0.0781187	1.4734	0.55917
325	1.34942	C41H74NO7P	0.0138829	3.5537	1.8293
224	1.33444	C28H46O4	-0.110406	1.1908	0.25197
330	1.31072	C41H81N2O6P	0.0285454	0.038618	-4.6946
453	1.30377	C46H87NO13	0.00679151	3.2327	1.6927
328	1.28636	C41H78NO7P	0.0192636	3.956	1.9841
332	1.25225	C41H83N2O6P	0.00976807	0.087405	-3.5161
393	1.22288	C44H84NO8P	0.0692114	1.2166	0.28289
403	1.21341	C42H80NO10P	-0.0204082	3.1076	1.6358
394	1.19293	C44H84NO8P	-0.0496091	1656.7	10.694
442	1.1891	C44H78NO10P	0.0243183	0.19357	-2.3691
317	1.15809	C39H79N2O6P	0.0243478	0.27906	-1.8413
444	1.14597	C46H78NO10P	0.0183461	0.24989	-2.0007
426	1.14081	C44H78NO10P	0.0239602	0.26222	-1.9311
183	1.13381	C21H40O4	-0.0157055	26.212	4.7122
348	1.11261	C41H80NO8P	0.00489484	2.3592	1.2383
413	1.08512	C44H84NO8P	0.02884	0.3009	-1.7326
353	1.0751	C43H78NO7P	-0.0213307	7.2723	2.8624
309	1.06295	C38H77N2O6P	0.0170117	0.3463	-1.5299
350	1.05236	C43H74NO7P	-0.0216739	2.6797	1.4221
424	1.05118	C47H91N2O6P	0.0147571	0.27115	-1.8828
390	1.04979	C44H82NO8P	-0.0207398	25.854	4.6923
261	1.04788	C26H54NO7P	0.0230413	0.21295	-2.2314

Supplemental Table 9. G1 metabolome compared to that of G2.
LC-MS analysis in positive ion mode
